# The impact of different care dependencies on people’s willingness to provide informal care: a discrete choice experiment in Germany

**DOI:** 10.1186/s13561-023-00448-5

**Published:** 2023-06-03

**Authors:** Lea de Jong, Torben Schmidt, Ann-Katrin Carstens, Kathrin Damm

**Affiliations:** Center for Health Economics Research Hannover (CHERH), Leibniz University Hannover, Hannover, Germany

**Keywords:** Willingness to care, Older adult care, Discrete choice experiment, Long-term care

## Abstract

**Background:**

Informal care provided by family members, friends, or neighbors is a major pillar in the German long-term care system. As the number of care-dependent older adults grow, ensuring their future care still relies on the willingness of family members, friends, or neighbors to assume the role of an informal caregiver. This study aimed to investigate the impact on people’s willingness to provide informal care to a close relative with predominately cognitive compared to physical impairments.

**Methods:**

An online survey was distributed to the general population in Germany, which resulted in 260 participants. A discrete choice experiment was created to elicit and quantify people’s preferences. A conditional logit model was used to investigate preferences and marginal willingness-to-accept values were estimated for one hour of informal caregiving.

**Results:**

Increased care time per day (hours) and expected duration of caregiving were negatively valued by the participants and reduced willingness to care. Descriptions of the two care dependencies had a significant impact on participants’ decisions. Having to provide care to a close relative with cognitive impairments was slightly preferred over caring for a relative with physical impairments.

**Conclusions:**

Our study results show the impact of different factors on the willingness to provide informal care to a close relative. How far the preference weights as well as the high willingness-to-accept values for an hour of caregiving can be explained by the sociodemographic structure of our cohort needs to be investigated by further research. Participants slightly preferred caring for a close relative with cognitive impairments, which might be explained by fear or discomfort with providing personal care to a relative with physical impairments or feelings of sympathy and pity towards people with dementia. Future qualitative research designs can help understand these motivations.

**Supplementary Information:**

The online version contains supplementary material available at 10.1186/s13561-023-00448-5.

## Introduction

When imagining how people would like to receive care when they get older, many aging adults prefer to remain in their familiar surroundings to maintain social ties and retain autonomy [1, 2]. Of a representative German sample, 87% stated that they would like to stay in their own homes when care-dependent in the future [3]. When family members, friends, or neighbors assume the role of a caregiver, they usually do not receive monetary compensation or payment. This so-called informal care is an essential pillar in long-term care systems worldwide and varies regarding type and intensity of help provided, location, and duration [4–6]. In 2019 in Germany, 80% of the 4.1 million care-dependent people were cared for in a home-based setting, either by family members, neighbors, or friends (56%) and/or by outpatient services (24%) [7]. Since the number of care-dependent older adults is expected to increase to six million by 2030 [7, 8], need for informal care is also expected to increase. Therefore, ensuring future care still relies on individuals’ willingness to assume the role of informal caregivers [4, 9]. However, surveys showed that younger respondents felt a lesser familial belonging and sense of responsibility toward caring for older relatives [10]. Additionally, close relatives’ ability to assume the role of informal caregivers has changed. Social changes, such as increased female labor force participation impact traditional caregiving. Moreover, the effects of globalization and increased job mobility often result in geographical distance between family members. Thus, the potential for informal care might decrease in the future, while demand grows [2].

Assuming the role of an informal caregiver potentially impacts, among other factors, the caregivers’ health, quality of life, occupation, and financial stability [8, 11]. Against the background that informal caregivers often experience high physical and mental strain, studies investigated the reasons or motivations for people to assume this time-consuming role [12, 13]. Zarzycki and Morrison (2021) understood willingness to care as a consequence of the underlying motivations for caregiving [14], such as feelings of love and affection, reciprocity, and a sense of obligation or feeling of indebtedness to the care receiver [12, 13, 15, 16]. Willingness to provide care is influenced by the potential caregiver’s socio-demographic factors (e.g. age, gender, family status, and place of residence), family structures and dynamics (e.g. number of siblings and proximity to children), religious affiliations, normative beliefs and values, and financial situation [14, 17, 18]. In addition, the type and severity of care dependency as well as the illness characteristics of the care receiver might influence a person’s willingness to provide care [14]. It enables certain predictions regarding the types of caregiving tasks required, duration of the caregiving situation, and potential intensity of changes as the illness progress [14, 19]. Although the actual duration and intensity of an informal caregiving situation are difficult to plan [13], certain factors that might influence a person’s willingness to provide care can be identified. However, these are understudied [14]. As an approximation, von dem Knesebeck et al. (2014) explored the emotional reactions and attitudes of the German general population toward people with dementia [20] and found that the majority expressed so-called pro-social reactions, they felt sympathy, pity, and wanted to help people with dementia. However, approximately 25% indicated that patients with dementia induced fear, and 46% felt uncomfortable in their presence. Furthermore, willingness to provide care was negatively associated with feelings of fear toward people with dementia, younger age, and lower socioeconomic status. Therefore, attitudes play a major role in a person’s willingness to provide care.

In informal care research, contingent valuation methods have been applied to explore the value of informal caregiving. Willingness-to-pay (WTP) values for a reduction of one hour of caregiving or willingness-to-accept (WTA) values for providing an additional hour of caregiving were estimated [21, 22]. Mentzakis et al. (2011) used a discrete choice experiment (DCE) to value informal care tasks, such as supervising, personal care, and household tasks [23]. Monetary compensation per informal care hour was also included to value informal care tasks [23]. To elicit people’s willingness to provide informal care in Germany, we conducted a postal survey of 280 participants in the general population using a DCE [24]. Data analysis revealed that increased hours of caregiving per day had the greatest negative impact on willingness to care. Monetary compensation could significantly increase willingness to provide care among individuals with a lower household income. As a central point of feedback from the postal survey, the type and severity of care dependency was found to be particularly relevant to know in advance and influenced people’s willingness to provide care. Especially, the issue of dementia was raised and mentioned as a potentially relevant factor compared to physical impairments. Therefore, it should be included in the DCE. To explore the relevance of the type of care dependency of a care receiver, this study further pursued the elicitation of the willingness to provide informal care among the German general population. This was done by adding two health descriptions of a hypothetical care receiver to the DCE with identical attributes and levels. Hence, the impact on participants’ willingness to care for a close relative with predominately cognitive compared to physical impairments was investigated.

## Methods

### DCE design

To quantify and elicit people’s preferences, a DCE was chosen as the central component of the survey. The research object was decomposed into a set of characteristics (attributes) and different levels. Hypothetical scenarios (choice sets) were designed with attributes and varied levels in each choice set. We created a symmetric experimental design with five quantitative attributes and three levels. The attributes and levels were chosen with the help of a systematic review [25] and semi-structured qualitative interviews [13, 26]. An overview of the identified attributes and levels, including their descriptions, is presented in Table 1.

**Table 1:** Overview of DCE attributes and levels

Attribute	Attribute description	Levels
Expected period of caregiving *(duration of care)*	The period of time the caregiver would care for and/or look after the person in need of care.	6 months 2 years 5 years
Care time *(hours per day)*	The amount of time (hours per day) the caregiver would provide care and/or supervise the person in need of care at home (e.g. personal care, household tasks, doctor visits etc.)	2 h per day 5 h per day 8 h per day
Formal care services *(frequency per week)*	The frequency of professional support that is additionally available to the caregiver (e.g. outpatient care services can assist with personal care or counsellors can help with any open questions). A visit lasts about 30 min.	None 3 to 4 times a week Daily
Respite *(weeks per year)*	The number of weeks a year that are available to the caregiver for a variety of respite options. During this time period, professionals care for the individual in need (e.g. during vacation).	None 3 weeks per year 6 weeks per year
Monetary compensation *(€ per hour)*	A wage replacement benefit (net) at the personal disposal of the caregiver. Paid as a financial compensation per hour for the care provided (in addition to the existing cash benefits by the LTC insurance in Germany).	€0 per hour €6 per hour €12 per hour

SAS software was used to create a D-efficient experimental design. In the design process, a series of designs was created and compared with the %MktEx and %ChoicEff macros and optimised in terms of its D-efficiency. D-efficiency is a standard measure of the goodness-of-fit that indicates how well the main effects can be estimated. A D-efficiency score of 1 is optimal, however all scores above 0.8 are considered reasonable [27]. The final experimental design was a fractional factorial design with 18 choice sets, blocked into three survey versions of six choice sets each to reduce respondents’ burden. The choice sets were checked for plausibility and randomly assigned to respondents. The design allowed for a clean estimation of all the main effects. Understandability of the attributes, levels, and description of health states and functionality of the online survey on different devices was piloted in a sample of the general population (n = 15).

### The survey

**Fig. 1:**
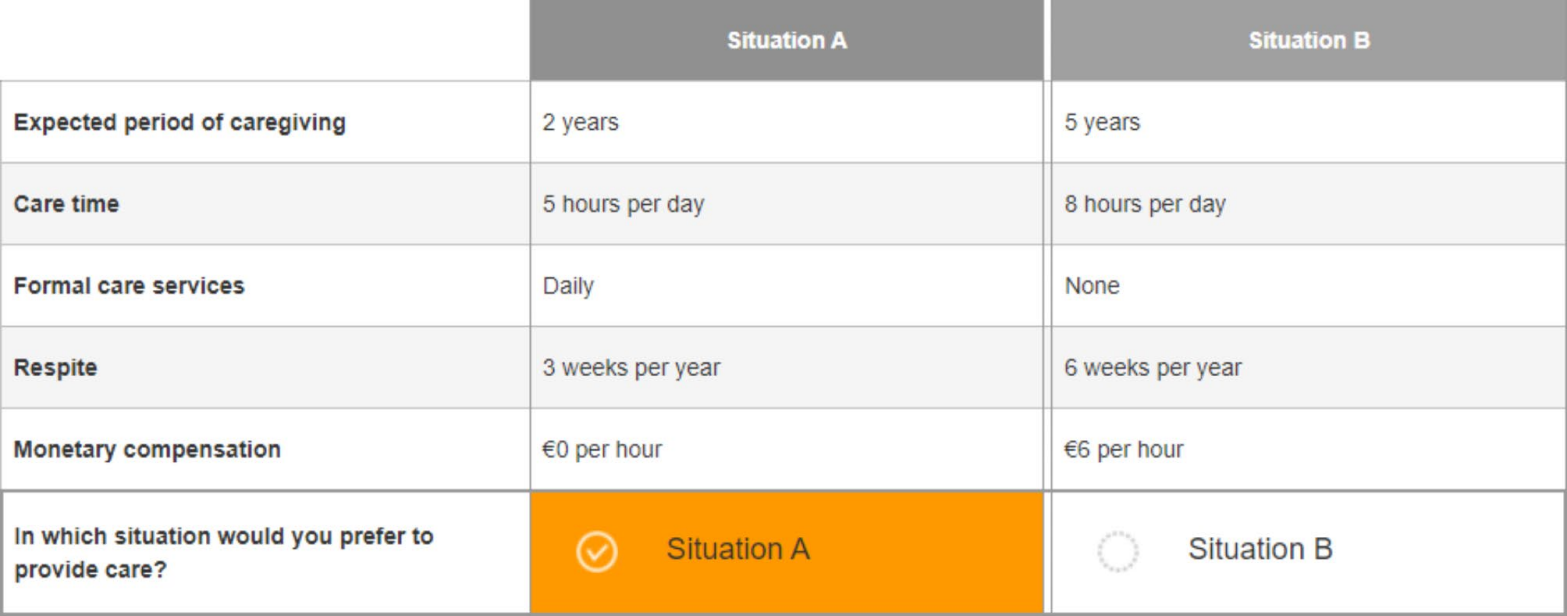
Example of a DCE choice set

An online survey was conducted using SurveyEngine [28]. Potential participants were informed that their answers would be collected anonymously and treated confidentially. No IP addresses were saved and collected data were only analyzed at Leibniz University and not transmitted to any third party. Following participants’ informed consent, the next two pages included instructions on how to complete the DCE-choice tasks. A detailed description of the attributes and levels, as well as an example choice set, is provided (see Fig. 1).

Identical to the previously performed postal study, the main research question in the online survey was “*Under what conditions are you willing to provide care to a close relative? What is important to you personally*?”. Respondents chose the preferred care situation (A or B) from the six choice sets depicted. For the choice sets, they were asked to imagine a close relative who could still be cared for in a home-based setting, while medical tasks would be cared for by professionals. To investigate the impact of elaborating on the type and severity of care-dependency of the hypothetical person, respondents were shown a first care dependency (*predominately physically impaired*) for the first three choice sets and a second care dependency (*predominately cognitively impaired*) for the others. Exact descriptions of the two care dependencies can be found in Table 2. The DCE choice sets were randomly assigned to the respondents, while ensuring that all choice sets were shown the same amount. Following the six DCE choice tasks, sociodemographic questions were posed (e.g., age, gender, family situation, and previous caregiving experience). Additionally, questions regarding the factors that influenced participant’s willingness to provide care were enquired. At the end, additional comments could be submitted to the research team.

**Table 2:** Description of the person in need of care (care dependency)

Care dependency of person #1
An older close relative of yours is in need of care. This person is severely physically impaired and needs assistance with personal hygiene, climbing stairs and walking. With assistive devices, the person can move around independently to a limited extent. Cognitively, the person is not impaired (no dementia). At times, night-time assistance is needed, as well as being accompanied to doctor’s visits and administering medication.
**Care dependency of person #2**
An older close relative of yours is in need of care. This person is barely limited in mobility and can still walk and climb stairs independently. However, he or she is becoming increasingly forgetful (dementia) and needs to be reminded about eating/drinking, personal hygiene and supported in the household. At times, you would have to help with personal hygiene or getting dressed. Partly, he/she is also very restless at night and confuses the day and night rhythm. Furthermore, he/she needs assistance with doctor’s visits and administering medication. Orientation outside his/her own home is difficult for him/her.

### Participants

The piloted survey was distributed to the general population in Germany through social media and flyers in public spaces (e.g. university notice boards) as well as doctors’ practices. Snowball sampling was used to increase the study population by asking the participants to share the survey link with other interested individuals. Individuals aged 18 to 65 years with no care dependency were eligible. We used Johnson and Orme’s formula (2003) and determined that the minimum required sample size was 125 respondents [29].

### Data analysis

Since the participants were forced to select survey responses, only fully completed surveys were included. Sociodemographic data and attitudinal questions were analyzed descriptively. For the choice data, participants’ discrete choices were analyzed using regression analysis to estimate the relative importance of each attribute. A conditional logit model (CLM) was used, and we assumed that the error terms were independently distributed with a Type 1 extreme value distribution [23, 30]. To determine the final CLM and assess the model fit, the Akaike (AIC) and Bayesian information criteria (BIC), log likelihood, and pseudo R-squared values were used. All analyses were conducted with R statistics version 4.0.4, using the package “survival” for CLM. For the multivariate analysis, all attribute levels were dummy coded and interpreted compared to the reference category, except for monetary compensation. Only statistically significant coefficients with a p-value of ≤ 0.05 could be interpreted. Since we included a cost attribute in the DCE choice tasks, we also calculated the marginal willingness-to-accept (MWTA) for attribute levels using the following equation:

$${MWTA}_{attribute}=-\left(\frac{{\beta }_{attribute}}{{\beta }_{cost attribute}}\right)$$

## Results

### Participant *characteristics*

In total, 260 individuals were included. An overview of the respondents’ characteristics is presented in Table 3. A considerably higher proportion were women (67%) and younger individuals (60%). Majority were unmarried (56%) and had no children (60%), which could be explained by the age structure. Of these, 64% had a household income of €1,500 or higher at their disposal. Half of the participants completed a university degree. Furthermore, 84% worked full-time, and the vast majority (90%) reported having a (very) good health status. Till date, 70% did not have any personal experience in organizing or providing informal care.

**Table 3:** Characteristics of included participants (n = 260)

	*N* = 260	
Sex		
Male	87	(33%)
Female	173	(67%)
Age group		
< 35 years	156	(60%)
>= 35 & <50 years	62	(24%)
> 50 years	42	(16%)
Marital status		
Single	146	(56%)
Married or in serious relationship	96	(37%)
Widowed	3	(1%)
Divorced or separated	15	(6%)
Having children		
Yes	103	(40%)
No	157	(60%)
Having siblings		
Yes	201	(77%)
No	59	(23%)
Education		
Completed primary education	69	(27%)
Completed vocational training	57	(22%)
Completed university degree	134	(52%)
Current employment status		
Part-time employment	28	(11%)
Full-time employment	219	(84%)
Unemployed	7	(3%)
Retired	6	(2%)
Household income		
Prefer not to say	16	(6%)
Below 500€ up to 1,500€	78	(30%)
1,500€ up to 3,000€	67	(26%)
3,000€ to 5,000€ and above	99	(38%)
Are your parents still alive?		
Yes, both	212	(82%)
One parent is deceased	29	(11%)
No	19	(7%)
Health status		
Very good	142	(55%)
Good	92	(35%)
Satisfactory	20	(8%)
Less good or bad	6	(2%)
Care experience		
Yes	79	(30%)
None	181	(70%)

### Results of the conditional logit model

Table 4 shows the preference weights for the CLM. All attributes were statistically significant, except for respite. Increased expected duration of caregiving and care time per day was negatively valued and therefore reduced willingness to provide care. The largest negative coefficient was found regarding providing eight compared to two hours of informal care per day. The MWTA for one hour of caregiving was €56.18 when providing care for eight compared to two hours per day. For an increased expected period of caregiving (duration), participants were willing to accept a MWTA of €9.26 and €37.98 per hour when caring for an expected period of two or five years compared to six months, respectively. The largest positive coefficient was found for daily formal care services supporting informal caregiving. Increased frequency of formal care services increased the odds of participants being willing to provide care by a factor of 2.8. Negative MWTA values indicated that participants were willing to waive monetary compensation or potentially pay for increased formal care services. Explanation of the care dependency before the DCE choice sets had a statistically significant impact on the respondents’ decisions. Providing care to a close relative with cognitive impairments was slightly preferred to caring for those with physical impairments.

**Table 4:** Conditional logit model (*main effects only*)

Attributes / levels	Coeff	OR	95% CI	SE	*p*-value	MWTA
Duration (*Ref: 6 months*)						
2 years	–0.332	0.718	(–0.577; − 0.086)	0.125	0.008*	9.26
5 years	–1.360	0.257	(–1.642; − 1.077)	0.144	0.000*	37.98
Care time (*Ref: 2 h/day*)						
5 h/day	–1.108	0.330	(–1.328; − 0.886)	0.113	0.000*	30.94
8 h/day	–2.011	0.134	(–2.239; − 1.783)	0.123	0.000*	56.18
Formal care services (*Ref: None*)						
3–4 times/week	0.358	1.430	(0.117; 0.599)	0.122	0.000*	–9.99
Daily	1.021	2.775	(0.782; 1.260)	0.122	0.000*	–28.51
Respite (*Ref: None*)						
3 weeks/year	–0.082	0.921	(–0.283; 0.119)	0.103	0.424	2.29
6 weeks/year	0.029	1.030	(–0.201; 0.260)	0.118	0.803	–0.82
Care dependency (*Ref: physical*)	0.220	1.246	(0.061; 0.378)	0.081	0.007*	6.14
Monetary compensation (*€/hour)*	0.036	1.037	(0.015; 0.057)	0.011	0.001*	
Log likelihood	– 1375.2					
Pseudo R^2^	0.21983					
No. of observations	3,120					
No. of coefficients	10					

Legend: Coeff = Coefficient, OR = Odds ratio, *significant at p < 0.05, SE = standard error, MWTA = marginal willingness to accept (€/hour), Ref = Reference category, Ref: physical = physically impaired

To further investigate the impact of explaining the two care dependencies beforehand, particularly the extent of preference heterogeneity, two additional CLMs were estimated and can be found in the Supplementary Material. Overall, the coefficients remained largely robust for all three CLMs. The main effect coefficients for the expected caregiving duration were similar. Specifically, increased duration of caregiving was valued negatively. However, providing care for five years instead of six months was valued as slightly worse when caring for a relative with cognitive impairments. The largest difference in coefficients was seen for providing care for five compared to two hours per day. Particularly, providing care for five hours to a relative with cognitive impairments was not valued as negatively, for which respondents were willing to accept €26.50 per hour compared to €43.77 per hour for a close relative with physical impairments. Another difference was the importance of formal care. For relatives with cognitive impairments, “formal care services” had a significant impact on respondents’ willingness to provide care, while for relatives with physical impairments, only daily formal care services had a significant impact.

## Discussion

This study aimed to build upon the previously performed DCE by de Jong et al. (2022) by investigating how an explicit depiction of a hypothetical type and severity of care dependency of a close relative impacted willingness to provide informal care. To ensure comparability, five identical attributes were used, previously shown to influence and establish relevance to a person’s willingness to provide care in a sample of the German general population. While all five attributes were found to be statistically significant and relevant in the postal sample, in our online sample, “respite” did not play a statistically significant role in respondent’s decision-making. Compared to the results of the CLM, “care time” constituted the most important aspect of caregiving for both samples and had a negative impact on people’s willingness. However, for the online sample, the need to provide more hours of informal care per day had a greater negative impact than the postal sample. In addition, differences in the importance of “formal care services” were identified. For the postal sample, both levels increased the odds of participants willing to care by approximately threefold. For the online sample, only daily formal care services compared to no services increased the odds of respondents being willing to care by a factor of 2.8. The MWTA values of both samples varied greatly, whereas the algebraic signs and directions were the same. The greatest difference was seen in the need to provide eight compared to two hours of care per day. While the postal sample would accept an hourly monetary compensation of €14.54 when providing eight hours of care, the online sample would accept a minimum of €56.18 per hour. In comparison, the current minimum wage in Germany is €12 [31]. In the postal survey, monetary compensation increased willingness to provide care among people with lower household income. In the online sample, significantly higher MWTA values were estimated, as monetary compensation did not have an important overall effect on participants’ willingness to provide care.

We suspect that the major differences in MWTA values and preference weights might be explained by differences in the sociodemographic structures of the two samples. While a similar percentage of women participated in both samples, the age structures differed greatly. The largest proportion of participants (60%) were younger than 35 years in the online sample compared to the largest proportion (48%) of 50 years and older in the postal sample. The age structure of the two samples also influenced the remaining sociodemographic variables. While majority of the postal sample were married (66%) and had children (68%), majority of the online sample were single (56%) and had no children (60%). Of the online sample, 84% were employed full-time during the survey compared to 48% of the postal sample. The majority of both the samples had a household income of €1,500 and above at their disposal; 38% of the online sample had a very high household income of €3,000 to €5,000 and above at their disposal compared to 20% of the postal sample. Furthermore, 90% of the online sample reported having a (very) good self-reported health status, compared to 65% of the postal sample. Only 30% of the online sample had personal experience in either organizing or providing informal care compared to nearly 60% of the postal sample. Younger age was found to be negatively associated with willingness to provide care in a previously conducted German survey [20], and younger respondents felt a lesser sense of responsibility toward caring for their older relatives [10]. In a previous qualitative study in Germany, younger respondents without any personal caregiving experience perceived aging and informal caregiving negatively, which influenced their willingness and motivation to provide informal care [13]. This negative perception of aging was also found in a comparative survey of young Germans and Americans. German participants generally viewed aging more negatively than the American respondents, and the results showed that cultural factors influenced the perceptions of aging, which included anxiety and fear associated with it [32]. Additionally, respite might not have played a statistically significant role in participants’ decisions in the online sample due to a higher proportion of individuals without caregiving experiences. Studies found that informal caregivers often identified respite services as an essential type of support [33, 34].

Depicting the type and severity of care dependency in advance had a statistically significant impact on participants’ willingness to provide informal care in the online sample. Taking care of relatives with cognitive impairments was slightly preferred over providing care to relatives with physical impairments. Two differences were observed when comparing the effects of describing the two care dependencies. First, formal care services were valued differently. When caring for a person with cognitive impairments, all forms of formal care support had a significant positive impact on participants’ willingness to care, which increased the odds by up to three times. When caring for a person with physical impairments, only daily formal care services had a significant impact, which increased the odds by 2.4. Second, providing care for five compared to two hours per day was not valued as negatively when caring for a person with cognitive impairments (Coeff: − 0.784) compared to those with physical impairments (Coeff: − 1.392). Providing care for eight hours instead of two had a similar impact on both care dependencies.

This was the first study to compare respondents’ willingness to provide care to a close relative either cognitively or physically impaired. Thus, a direct comparison with similar studies was difficult. Providing care to a relative with cognitive impairments was slightly preferred in our sample; one possible explanation might be discomfort of providing personal care to a relative who was physically impaired. For our sample, having formal care services assisted by personal care was the most important (mean: 3.68, median: 4.00) compared to household tasks (mean: 2.01, median: 2.00) or assistance with organizing everyday life (mean: 2.07, median: 2.00). In Mentzakis et al.’s study (2011), informal care tasks were valued, and personal care was the most influential (negatively valued) attribute [23]. In contrast, as von dem Knesebeck et al.’s study (2014) showed, majority of German respondents felt sympathy or pity for people with cognitive impairments, such as dementia, and felt the need to help them [20].

### Limitations

Since this survey was conducted online, a very young cohort of respondents participated. Therefore, the results should be interpreted cautiously and cannot be generalized to the entire German general population. While we compared our online results with the postal survey regarding the overall impact of the five attributes, the effect of including a description of the type and severity of care dependency was tested only in the online survey. Furthermore, temporal changes could not be investigated since two different samples participated in the surveys. In an ideal research scenario, an identical sample or a sample similar in socio-demographic variables to the postal survey would have been given the online choice sets, which included the two descriptions of the care dependencies. Thus, the sole impact of describing either a cognitively or physically impaired relative could be estimated. In addition, we do not know what respondents specifically associated with the depicted health states, meaning which motivations and feelings a cognitive versus physically impaired person were brought out. Future qualitative research designs can help understand these motivations.

The survey was distributed through social media and flyers. Due to this arbitrary sampling (self-selection of participants), the analysis of non-responders and the possibility of correcting non-response is impeded. Refusal or participation are particularly dependent on the subject of the survey.

Choice experiments enable the preference elicitation of a large sample using trade-off decisions. The hypothetical scenarios allow the inclusion of both people with and without caregiving experience (prospectively). Thus, a decision-making situation determining the general willingness or availability of informal caregivers can be simulated. An alternative approach would be to survey individuals with experience in informal caregiving. Such an approach could focus more on insights, experiences, or challenges but would exclude individuals with no experience in informal care who may need to make similar decisions in the future.

## Conclusion

This study investigated people’s willingness to provide informal care in the German general population using a DCE. The impact of participants’ willingness to care for a relative with cognitive versus physical impairments was investigated. Participants slightly preferred caring for a close relative with cognitive impairments, which might be explained by fear or discomfort with providing personal care to a relative with physical impairments or feelings of sympathy and pity towards people with dementia. Increased care time per day had the greatest negative impact on participants’ willingness to provide care, for which respondents were willing to accept a minimum of €56.18 per hour of caregiving when providing care for eight compared to two hours per day. Younger age and lack of personal experience in caregiving might explain the preference weights and high MWTA values in this survey, as younger age was found to be negatively associated with willingness to provide care in other studies.

## Electronic supplementary material

Below is the link to the electronic supplementary material.

Supplementary Material 1

## Data Availability

The datasets generated during the current study are not publicly available, as the publication of the collected primary data is not covered by the informed consent, but are available from the corresponding author upon reasonable request.
