# Supplementary material for: The impact of different care dependencies on people’s willingness to provide informal care: a discrete choice experiment in Germany
Source: Health Econ Rev. 2023 Jun 3;13:35. doi: 10.1186/s13561-023-00448-5 (PMC10239163; doi:10.1186/s13561-023-00448-5)
Supplement: Supplementary file 1 — Supplementary Material 1 [file 13561_2023_448_MOESM1_ESM.docx]

**Supplementary material**

Table 1: Conditional logit model (*main effects only, health status: physical impairment*)

| **Attributes / levels** | **Coeff** | **OR** | **95% CI** | **SE** | ***p*-value** | **MWTA** |
| --- | --- | --- | --- | --- | --- | --- |
| Duration (*Ref: 6 months*) |  |  |  |  |  |  |
| 2 years | –0.375 | 0.688 | (–0.744; –0.005) | 0.188 | 0.047* | 11.78 |
| 5 years | –1.385 | 0.250 | (–1.815; –0.954) | 0.220 | 0.000* | 43.54 |
| Care time (*Ref: 2 hours/day*) |  |  |  |  |  |  |
| 5 hours/day | –1.392 | 0.249 | (–1.696; –1.088) | 0.155 | 0.000* | 43.77 |
| 8 hours/day | –1.969 | 0.140 | (–2.282; –1.656) | 0.160 | 0.000* | 61.90 |
| Formal care services (*Ref: None*) | |  |  |  |  |  |
| 3–4 times/week | 0.235 | 1.265 | (–0.099; 0.569) | 0.170 | 0.168 | –7.38 |
| Daily | 0.872 | 2.391 | (0.556; 1.187) | 0.161 | 0.000* | –27.41 |
| Respite (*Ref: None*) |  |  |  |  |  |  |
| 3 weeks/year | –0.043 | 0.958 | (–0.316; 0.231) | 0.140 | 0.759 | 1.35 |
| 6 weeks/year | 0.181 | 1.199 | (–0.124; 0.485) | 0.155 | 0.245 | –5.69 |
| Monetary compensation (*€/hour)* | 0.032 | 1.032 | (–0.001; 0.064) | 0.017 | 0.054 |  |
|  |  |  |  |  |  |  |
| Log likelihood | –591.88 | |  |  |  |  |
| Pseudo R^2^ | 0.22854 | |  |  |  |  |
| AIC | 1201.8 |  |  |  |  |  |
| BIC | 1243.7 |  |  |  |  |  |
| No. of observations | 1560 |  |  |  |  |  |
| No. of coefficients | 9 |  |  |  |  |  |

*Legend: OR = Odds ratio, *significant at p<0.05, AIC = Akaike information criteria, BIC = Bayesian information criteria, SE = standard error, MWTA = marginal willingness to accept (€/hour), Ref = Reference category*

Table 2: Conditional logit model (*main effects only, health status: cognitive impairment*)

| **Attributes / levels** | **Coeff** | **OR** | **95% CI** | **SE** | ***p*-value** | **MWTA** |
| --- | --- | --- | --- | --- | --- | --- |
| Duration (*Ref: 6 months*) |  |  |  |  |  |  |
| 2 years | –0.440 | 0.644 | (–0.822; –0.057) | 0.195 | 0.024* | 14.86 |
| 5 years | –1.488 | 0.226 | (–1.897; –1.078) | 0.209 | 0.000* | 50.26 |
| Care time (*Ref: 2 hours/day*) |  |  |  |  |  |  |
| 5 hours/day | –0.784 | 0.456 | (–1.106; –0.463) | 0.164 | 0.000* | 26.50 |
| 8 hours/day | –1.990 | 0.137 | (–2.349; –1.632) | 0.183 | 0.000* | 67.24 |
| Formal care services (*Ref: None*) | |  |  |  |  |  |
| 3–4 times/week | 0.490 | 1.633 | (0.122; 0.859) | 0.188 | 0.009* | –16.57 |
| Daily | 1.126 | 3.083 | (0.761; 1.491) | 0.186 | 0.000* | –38.04 |
| Respite (*Ref: None*) |  |  |  |  |  |  |
| 3 weeks/year | 0.036 | 1.037 | (–0.281; 0.353) | 0.162 | 0.824 | –1.21 |
| 6 weeks/year | 0.127 | 1.135 | (–0.228; 0.482) | 0.181 | 0.483 | –4.29 |
| Monetary compensation (*€/hour)* | 0.030 | 1.030 | (–0.000; 0.059) | 0.015 | 0.053 |  |
|  |  |  |  |  |  |  |
| Log likelihood | –557.5 | |  |  |  |  |
| Pseudo R^2^ | 0.27268 | |  |  |  |  |
| AIC | 1133 |  |  |  |  |  |
| BIC | 1174.9 |  |  |  |  |  |
| No. of observations | 1560 |  |  |  |  |  |
| No. of coefficients | 9 |  |  |  |  |  |

*Legend: OR = Odds ratio, *significant at p<0.05, AIC = Akaike information criteria, BIC = Bayesian information criteria, SE = standard error, MWTA = marginal willingness to accept (€/hour), Ref = Reference category*
